# Perceptual learning of a crowding task: Effects of anisotropy and optotype

**DOI:** 10.1167/jov.21.11.13

**Published:** 2021-10-21

**Authors:** Tina Plank, Laura Lerner, Jana Tuschewski, Maja Pawellek, Maka Malania, Mark W. Greenlee

**Affiliations:** 1 Institute for Experimental Psychology, University of Regensburg, Regensburg, Germany; 2 Children's University Hospital, University of Regensburg, Regensburg, Germany

**Keywords:** crowding, perceptual learning, psychophysics, visual training, peripheral vision

## Abstract

Visual crowding refers to the impairment of recognizing peripherally presented objects flanked by distractors. Crowding effects, exhibiting a certain spatial extent between target and flankers, can be reduced by perceptual learning. In this experiment, we investigated the learning-induced reduction of crowding in normally sighted participants and tested if learning on one optotype (Landolt-C) transfers to another (Tumbling-E) or vice versa. Twenty-three normally sighted participants (18–42 years) trained on a crowding task in the right-upper quadrant (target at 6.5 degrees eccentricity) over four sessions. Half of the participants had the four-alternative forced-choice task to discriminate the orientation of a Landolt-C, the other half of participants had the task to discriminate the orientation of a Tumbling-E, each flanked by distractors. In the fifth session, all participants switched to the other untrained optotype, respectively. Learning success was measured as reduction of the spatial extent of crowding. We found an overall significant and comparable learning-induced reduction of crowding in both conditions (Landolt-C and Tumbling-E). However, only in the group who trained on the Landolt-C task did learning effects transfer to the other optotype. The specific target-flanker-constellations may modulate the transfer effects found here. Perceptual learning of a crowding task with optotypes could be a promising tool in rehabilitation programs to help improve peripheral vision (e.g. in patients with central vision loss), but the dependence of possible transfer effects on the optotype and distractors used requires further clarification.

## Introduction

Visual crowding refers to the impairment in the ability to recognize peripherally presented targets flanked by distractors (e.g. Whitney & Levi, 2011). There are several studies showing improved recognition performance for flanked stimuli (a decrease of the crowding effect) after training (e.g. Chung, 2007; Huckauf & Nazir, 2007; Hussain, Webb, Astle, & McGraw, 2012; Maniglia, Pavan, Cuturi, Campana, Sato, & Casco, 2011; Sun, Chung, & Tjan, 2010; Xiong, Yu, & Zhang, 2015; Yashar, Chen, & Carrasco, 2015), and some studies have also looked into possible transfer effects of training and the specific role of target-flanker-combinations. For example, Chung (2007) used trigrams of letters with different spacings that were presented at 10 degrees eccentricity in the lower visual field, and found a significant improvement in performance for recognizing the middle target letter through training. This training effect transferred to other, nontrained, letter spacings, indicating that both the strength of the crowding effect and the size of the crowding zone decreased as a result of the training. The latter could be explained by shrinking receptive fields representing the trained area in visual cortex. Chung (2007) suspected the location of such neural populations in V2. Huckauf and Nazir (2007) investigated stimulus-specific learning of crowding. Their results showed that learning effects only occurred when the target-flanker combination did not change during training, and thus a better discrimination between flanker and target could be learned. The authors also probed whether there was a difference in learning between meaningless series of letters and words. In general, isolated letters were better recognized than flanked letters, whereby those flanked letters that were part of a meaningful word were better recognized. Yeotikar, Khuu, Asper, and Suttle (2013) used a contrast discrimination task to investigate perceptual learning and a possible transfer effect in crowding. Target and flankers were Gabor patterns that were either collinearly oriented (iso-oriented [ISO]), or the pattern to be identified was presented rotated 90 degrees (CROSS) with respect to the flankers. In addition, a control group was presented with the target without flankers to control for general perceptual learning. On each trial, participants saw a stimulus constellation above and below the fixation point and were asked to compare the two targets with respect to their contrast. Before and after training, participants were tested at different eccentricities and flanker distances. The training itself took place at the lowest flanker spacing (0.64 degrees) and the highest eccentricity (9 degrees; i.e. under the most difficult conditions). Comparing pre- and post-test, a perceptual learning effect occurred for all three conditions (ISO, CROSS, and control), but was greatest for the ISO condition. Transfer to untrained flanker spacing, eccentricity, or stimulus configuration (e.g. to CROSS when ISO was trained) did not occur here with the Gabor stimuli. Yeotikar and colleagues (2013) concluded that the learning effect was due to more efficient processing of the target-flanker combination. Yashar et al. (2015) also tested transfer effects in a learning experiment on crowding where they used combinations of letters as targets and flankers. They found that learning was location specific and did not transfer to the other visual hemifield. In a second experiment, the authors varied target and flanker polarity and found a learning effect only for the condition with same flanker polarity. Transfer effects in training of crowding thus appear to depend to a certain extent on the specific target-and-flanker constellations.

The spatial shape of the crowding zone in the peripheral visual field is characterized by a radial-tangential anisotropy (i.e. the crowding zone has an elliptical shape that is elongated along the radial axis; Toet & Levi, 1992; Whitney & Levi, 2011), resulting in stronger crowding for radially arranged flankers in contrast to tangentially arranged flankers. Interestingly, Chung (2013) found, in patients with central vision loss, who develop a kind of pseudo-fovea at intact peripheral retina called the preferred retinal locus (PRL), that the crowding area around the PRL did not show this elliptical shape but rather had a circular shape, resembling the shape of the foveal crowding area found in normally sighted persons. On a neuronal level, Kwon, Bao, Millin, and Tjan (2014) could demonstrate the presence of a radial-tangential anisotropy in V1 that is manifested in BOLD signal suppression for radially arranged flanker conditions compared to tangentially arranged flanker conditions. It was established that stronger crowding produces a more pronounced reduction in the BOLD signal (Millin, Arman, Chung, & Tjan, 2014). In a recent experiment, we asked to which extent crowding, and specifically the radial-tangential anisotropy in crowding, could be reduced or even eliminated by training and how improvements in performance were reflected in neural responses (Malania, Pawellek, Plank, & Greenlee, 2020). For this purpose, a group of normally sighted healthy volunteers were trained on a crowding task with the optotype Landolt-C as target stimulus flanked by same-sized rings, and behavioral training was accompanied by two functional magnetic resonance imaging (fMRI) sessions (pre- and post-training). We found that the spatial extent of crowding decreased in the course of training and this reduction in crowding was more prominent along the radial axis (i.e. leading to a more circular shape of the crowding zone). The BOLD signal also changed: prior to training, participants showed, on average, a suppressed BOLD activity in the condition with target-present and radial flankers while we observed a higher BOLD signal after training compared to the target-absent-radial flankers’ condition (i.e. when only radially arranged flankers were presented). Those results suggested that changes in BOLD responses after training reflect training-induced reduction of crowding as well as training-induced plasticity in early visual cortex (Malania et al., 2020).

In the present study, our goal was twofold. First, we aimed to replicate our findings from Malania et al. (2020), where we found a reduction of crowding and a reduction of the radial-tangential anisotropy through perceptual learning on the target optotype Landolt-C, in another subject group, and to see if similar results could be obtained for other optotypes, such as the Tumbling-E. Second, we wanted to probe transfer effects of learning from one optotype to the other to see if optotypes are interchangeable in this regard. As previous studies have shown, the target-flanker-constellation plays an important role in transfer effects. Here, we aimed to keep the respective flankers as similar as possible to their target stimuli. We used closed rings as flankers for the Landolt-C targets and closed squares with a crossbar as flankers for the Tumbling-E target. A transfer of learning to other optotypes would be a sign that crowding could be generally reduced by this task, pointing to possible benefits by such a training for people who are reliant on their peripheral visual fields like patients with central vision loss (e.g. due to macular degeneration).

## Methods

### Participants

Overall, 30 participants completed the experiment, but the data of seven participants had to be excluded, in two cases because of missing data due to technical issues and in five cases due to inadequate fit of psychometric functions (R^2^ <0.1; see Supplementary Materials for a full description [hit rates] of all of the data collected in our study). Thus, the data of 23 participants entered the group analysis (age range = 18–42 years; mean age = 22.8 years; 4 men and 19 women). All participants reported normal or corrected to normal vision and gave written informed consent prior to participation. All participants were students of the University of Regensburg. No monetary compensation was provided, but the students received course credit for participating. The experimental procedures were approved by the Ethics Commission of the University of Regensburg and conducted in accordance with the ethical guidelines of the Declaration of Helsinki.

### Apparatus, stimuli, and procedure

The experimental setup paralleled the one used in Malania et al. (2020), but due to the coronavirus disease 2019 (COVID-19) pandemic it was not possible to test all participants in our laboratory. Seventeen subjects whose data were included in the analysis completed the task in our laboratory, the remaining six subjects performed the experiment at home at their own computers. In the laboratory, stimuli were presented on a 19 inch LCD monitor (Dell; resolution: 1024 × 768 pixels) at a viewing distance of 54 cm, which was ensured by the use of a chinrest. For the experiments at home, participants were instructed to measure the distance from the fixation cross to the target stimulus on their screens and to calculate the correct individual viewing distance by use of a Microsoft Excel sheet provided to them. These subjects also provided the size and resolution of their screens, so that it was possible to ensure that all stimuli were visible and no flankers were cut off. The experiment in the laboratory was conducted in a dimly lit room, and the participants at home were instructed to use a comparable room lighting.

**Figure 1.**
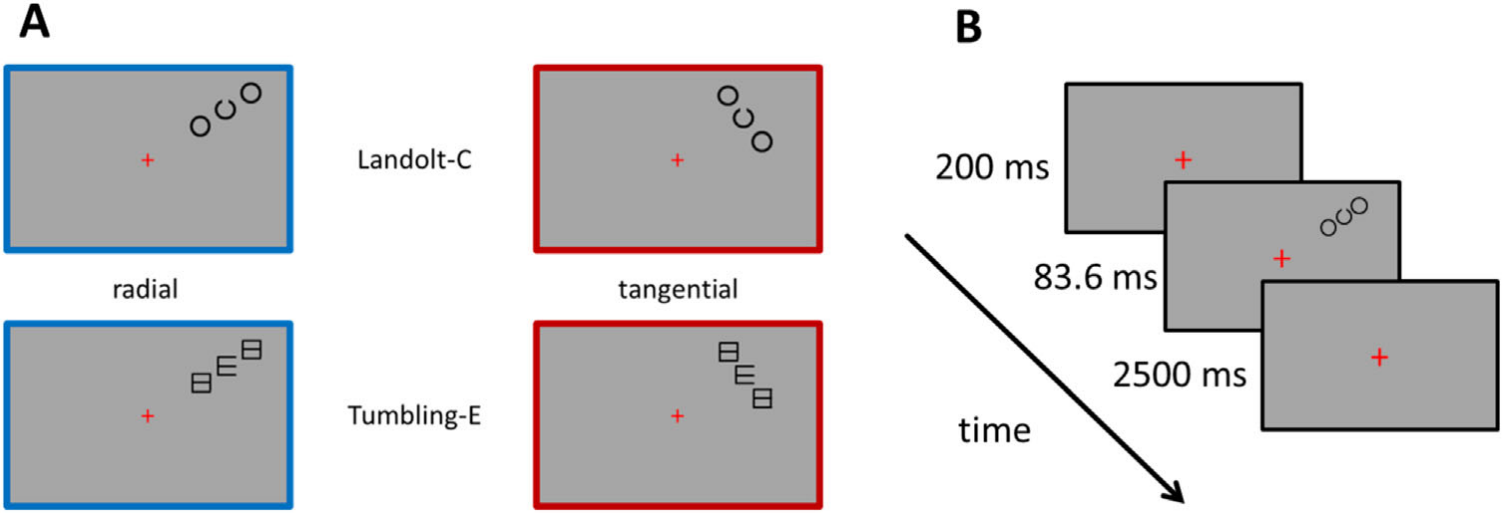
Schematic depiction of stimuli arrangements and stimulus sequence. (**A**) Radial (with blue frame) and tangential (with red frame) stimulus arrangements for the Landolt-C and the Tumbling-E optotype, respectively. The colored frames are used for illustration purposes only. (**B**) Timeline of one trial in the Landolt-C task.

To keep conditions equal to the setup in Malania et al. (2020), all participants trained on a crowding task in the right-upper quadrant. Target stimuli were black optotypes (Landolt-C and Tumbling-E) presented on a uniform grey background at 6.5 degrees visual angle eccentricity and at an angle of 25 degrees clockwise from the vertical meridian. Target size was 0.75 degrees visual angle. Targets were flanked by same-sized distractors (closed rings or squares with a crossbar, respectively) that were placed radially or tangentially with respect to the fovea (see Figure 1A). Dimensions were round and squared, respectively, for targets and flankers alike. Landolt-Cs and Tumbling-Es were designed according to standard dimensions for optotypes, as for example provided in the Freiburg Visual Acuity and Contrast Test (FrACT; Bach, 1996), with the gap and sign/stroke width equal to one fifth of the diameter/edge length. The center-to-center distance of the target-flanker-arrangements varied between 0.75, 1, 1.5, 2, 2.5, and 3 degrees visual angle, using the method of constant stimuli. In an additional control condition, the target stimulus was shown without flankers.

Stimuli were generated and controlled by the software Presentation (Neurobehavioral Systems; www.neurobs.com). On each trial, the task was to indicate the direction of the opening of the Landolt-C/Tumbling-E (4AFC: left, right, up, or down) by pressing the respective arrow key on the computer keyboard. The timeline of one trial as conducted in the lab is depicted in Figure 1B. The target stimuli were shown for 83.6 ms. With the six subjects who did the experiment at home, target duration deviated slightly with 83.3 ms for five subjects and 100 ms for one subject due to the technical configuration of their own monitors. Participants were instructed to maintain central fixation at the fixation cross throughout the experiment. During the laboratory experiment, fixation was monitored by use of a video eye-tracker (Cambridge Research Systems, CRS Ltd.). The six subjects who did the experiment at home had an additional fixation task to monitor their central fixation. During 84 trials in each session, which were randomly interleaved, instead of the crowding task the letters O or D (size = 0.53 degrees visual angle) were presented centrally in random order and the participants had to indicate by button press, which one of the two letters was presented. All six participants performed that task well (over 80% correct in all sessions).

In each session, participants in the laboratory completed two blocks (one in the radial and one in the tangential condition, respectively) with 392 trials each (56 in each flanker condition). The order of radial and tangential blocks was counterbalanced over subjects. Participants, who did the experiment at home, had to conduct the additional 84 trials of the fixation task. Therefore, the two blocks (radial and tangential) were split into four (two radial and two tangential). For one of those participants in one session (session 3), only half of the trials in the radial condition could be analyzed due to loss of data. No trial-by-trial feedback was given, but after each block the number of hits was provided to the subjects. Each session was performed on a different day. There was a maximum gap of 4 days between two sessions. Participants trained either the Landolt-C task or the Tumbling-E task for four sessions and switched to the other optotype in session 5. Eleven participants (4 at home) trained the Landolt-C task and 12 participants (2 at home) trained the Tumbling-E task.

### Data analysis

For each session, flanker condition and subject, proportions of correct responses were calculated out of the data. The proportions correct for the flanker spacings 0.75, 1, 1.5, 2, 2.5, and 3 degrees visual angle were then transformed into z-scores, and a psychometric function was fit as a cumulative normal function to the z-scores by linear regression (Gescheider, 1997) for each subject, session and flanker configuration (radial, tangential). Where z-scores were not defined, for example in cases with a proportion correct of 1, z-scores were approximated by applying a method described in Wickens (2002), (i.e. by subtracting ½ observation from the correctly identified number of trials), resulting in an approximated proportion correct of 0.99. The spatial extent of crowding was then estimated as the 62.5% accuracy thresholds from the psychometric functions. Learning success was measured as reduction of this spatial extent of crowding. Group analyses were performed by the use of analyses of variance (ANOVAs) and *t*-tests. In cases where the sphericity assumption was violated in ANOVAs, we applied Huynh-Feldt correction of degrees of freedom.

## Results

### No-flanker control condition

Table 1 shows the mean proportions of correct responses in the no-flanker condition for the Landolt-C and Tumbling-E optotypes that served as a control condition to ensure that orientations of both optotypes are easily recognized when they are presented at the chosen eccentricity of 6.5 degrees visual angle without flankers. At first, we tested if the nuisance variable testing locality (experiment done in the laboratory or at home) had a significant influence on the outcome. To this end, we applied *t*-tests for independent samples to each variable (session 1 to 5) to determine any significant differences between those two groups (laboratory versus home). For all variables we obtained no significant differences between the two testing localities (all *p* > 0.05). Subsequently, a repeated-measures ANOVA with the within-subjects factor session (session 1–5) and the between-subjects factor optotype (Landolt-C versus Tumbling-E) was conducted and revealed a marginally significant main effect of session (F [3.05, 64.03] = 2.4; *p* = 0.074; η^2^ = 0.10; Huynh-Feldt corrected), but neither a significant main effect of optotype (F [1, 21] = 0.31; *p* = 0.58; η^2^ = 0.015) nor a significant interaction between session and optotype (F [3.05, 64.03] = 0.93; *p* = 0.43; η^2^ = 0.04; Huynh-Feldt corrected).

**Table 1.** Mean values and standard errors (in parentheses) for the proportions correct of identifying the direction of the target stimuli Landolt-C and Tumbling-E in the control condition without flankers.

	Session 1	Session 2	Session 3	Session 4	Session 5 (Transfer to other optotype)
**Training of Landolt-C**	0.97 (0.008)	0.99 (0.004)	0.99 (0.002)	0.98 (0.012)	0.99 (0.002)
**Training of Tumbling-E**	0.98 (0.007)	0.99 (0.002)	0.99 (0.005)	0.99 (0.004)	0.99 (0.003)

### Perceptual learning of crowding

Supplementary Figure S1 shows the trends of the mean proportions of correct responses for each flanker spacing, condition, and group in the course of the training sessions. A repeated-measures ANOVA with the within-subjects factors session (session 1–4), flanker configuration (radial and tangential) and flanker spacing (0.75, 1, 1.5, 2, 2.5, and 3) and the between-subjects factor optotype (Landolt-C versus Tumbling-E) revealed a significant main effect of session (F [2.1, 44.6] = 34.8; *p* < 0.001; η^2^ = 0.62; Huynh-Feldt corrected), a significant main effect of flanker configuration (F [1, 21] = 18.7; *p* < 0.001; η^2^ = 0.47) and a significant main effect of flanker spacing (F [3.4, 70.6] = 371.3; *p* < 0.001; η^2^ = 0.95; Huynh-Feldt corrected). Additionally, the interactions between session and flanker configuration (F [2.5, 51.7] = 3.3; *p* = 0.033; η^2^ = 0.14; Huynh-Feldt corrected), between flanker configuration and flanker spacing (F [2.7, 56.0] = 7.8; *p* < 0.001; η^2^ = 0.27; Huynh-Feldt corrected), and between session and flanker spacing (F [15, 315] = 6.2; *p* < 0.001; η^2^ = 0.23), as well as the three-way interaction session times flanker configuration times flanker spacing (F [13.1, 274.4] = 1.8; *p* = 0.041; η^2^ = 0.08; Huynh-Feldt corrected) were significant. The group main effect optotype turned out to be not significant (F [1, 21] = 0.83; *p* = 0.373; η^2^ = 0.04), as well as the interactions session times optotype (F [2.1, 44.6] = 0.18; *p* = 0.847; η^2^ = 0.01; Huynh-Feldt corrected), flanker configuration times optotype (F [1, 21] = 0.29; *p* = 0.597; η^2^ = 0.01), flanker spacing times optotype (F [3.4, 70.6] = 0.88; *p* =.0486; η^2^ = 0.04; Huynh-Feldt corrected), session times flanker configuration times optotype (F [2.5, 51.7] = 0.54; *p* = 0.622; η^2^ = 0.02; Huynh-Feldt corrected), flanker configuration times flanker spacing times optotype (F [2.7, 56.0] = 1.4; *p* = 0.240; η^2^ = 0.06; Huynh-Feldt corrected), session times flanker spacing times optotype (F [15, 315] = 1.3; *p* = 0.197; η^2^ = 0.06) and session times flanker configuration times flanker spacing times optotype (F [13.1, 274.4] = 0.90; *p* = 0.552; η^2^ = 0.04; Huynh-Feldt corrected).

**Figure 2.**
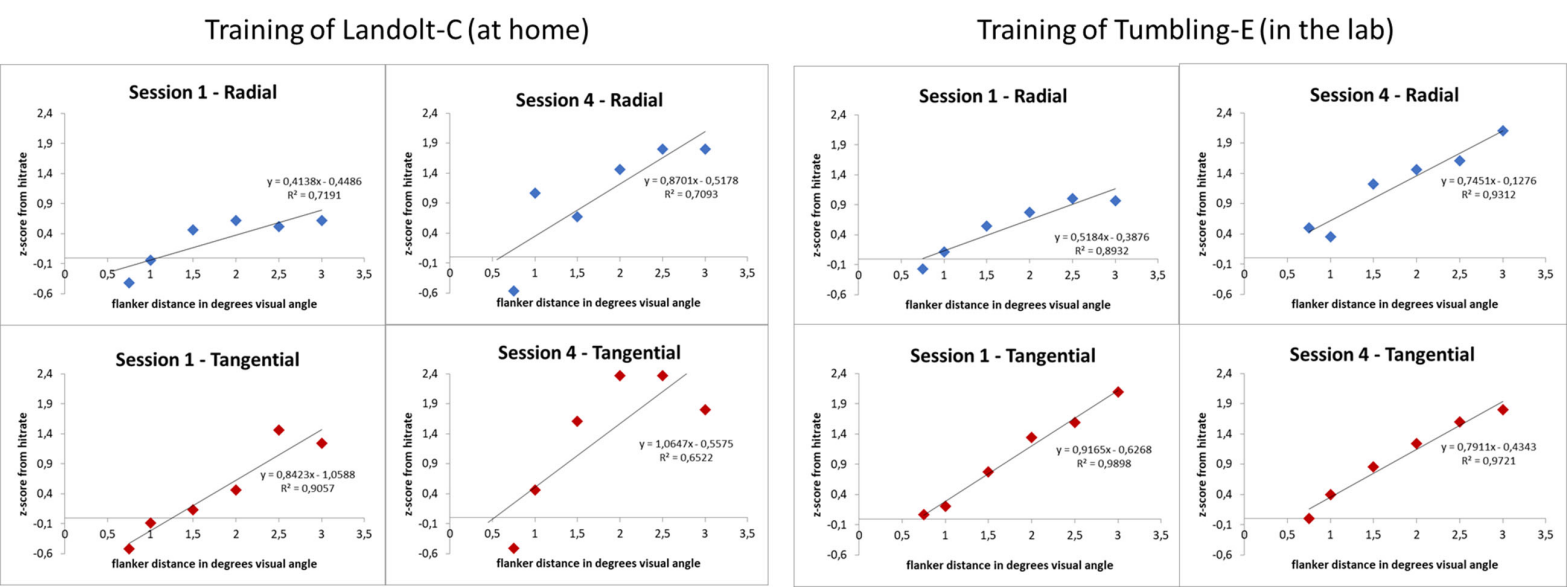
Examples of psychometric functions of two subjects fit by linear regression to the z-scores calculated from hit rates for pre- (session 1) and post-training (session 4) for the radial condition (upper row, in blue) and the tangential condition (lower row, in red). Left panel: Data from one subject who trained the Landolt-C task at home. Right panel: Data from one subject who trained the Tumbling-E task in the laboratory.

We estimated the spatial extent of crowding as 62.5% correct thresholds by adjusting psychometric functions to the individual data (see above). Figure 2 shows examples of psychometric functions from one subject who performed the Landolt-C training at home (left panel) and from one other subject who performed the Tumbling-E training in the laboratory (right panel). Mean R^2^ were calculated as goodness-of-fit measures for all conditions and sessions, lying between 0.72 (SE = 0.07) in session 1 of the radial condition for the optotype Tumbling-E and 0.92 (SE = 0.02) in session 1 of the tangential condition for the optotype Tumbling-E.

**Figure 3.**
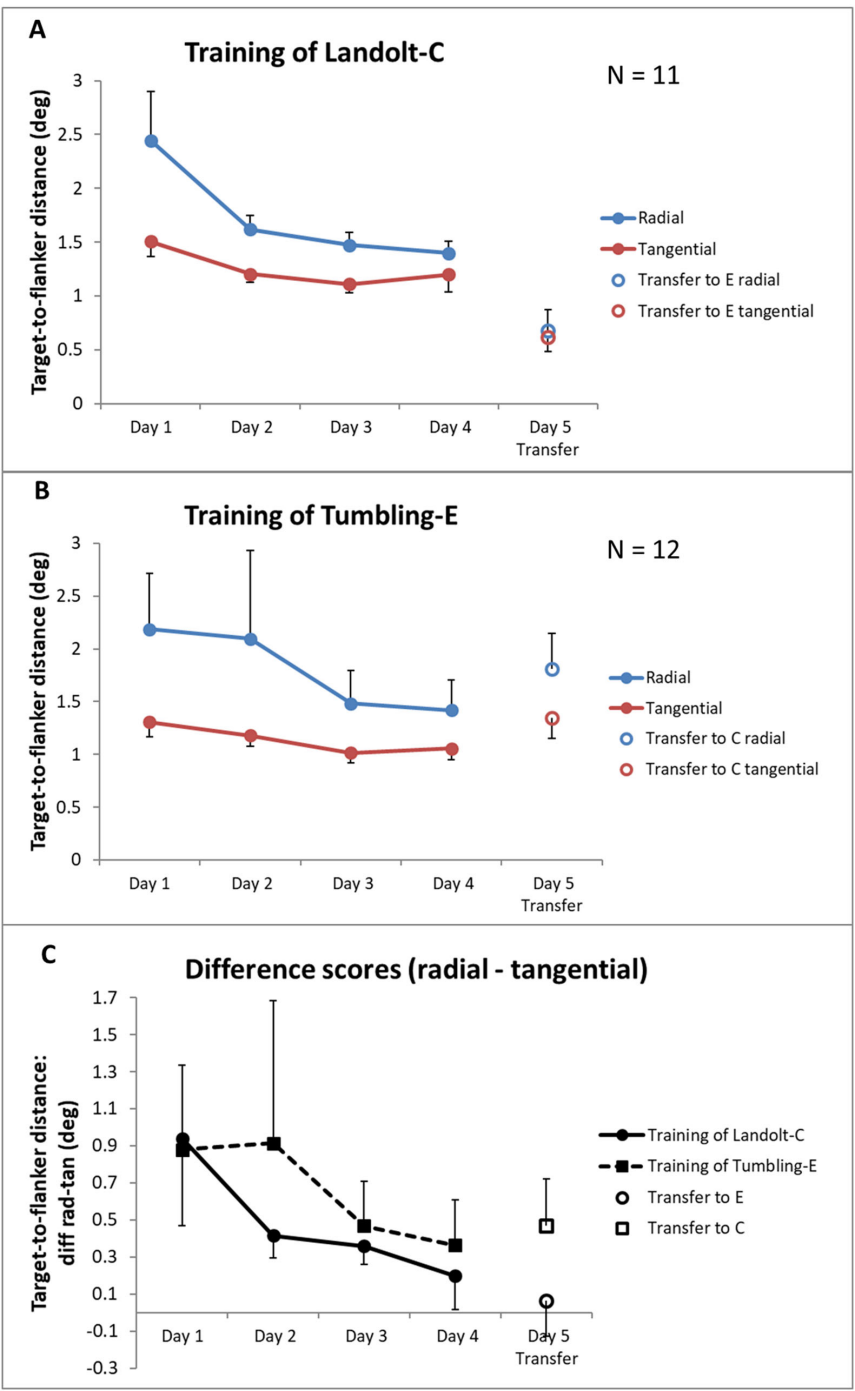
Mean target-to-flanker distances (62.5% thresholds) (±1 SE) over the training period of four days, as well as for the transfer to the other optotype on day 5. (**A**) Group who trained with Landolt-C and transferred to Tumbling-E on day 5. (**B**) Group who trained with Tumbling-E and transferred to Landolt-C on day 5. The data for the radial flanker configurations are shown in blue, the data for the tangential flanker configurations are shown in red. (**C**) Mean differences between target-to-flanker distances in the radial and tangential conditions, solid line and circles for the group who trained with Landolt-Cs, dashed line and squares for the group who trained with Tumbling-Es.

The target-to-flanker distances (62.5% thresholds) as functions of training sessions are depicted in Figure 3. For statistical analyses, we first checked again for the influence of the nuisance variable testing locality (laboratory versus home) with *t*-tests for independent samples for each variable. Again, we obtained no significant differences between the two localities (all *p* > 0.05).

Training effects over the initial 4 days were then analyzed by use of a repeated measures ANOVA with the within-subjects factors session (sessions 1–4) and flanker configuration (radial versus tangential) and the between-subjects factor optotype (Landolt-C versus Tumbling-E). We determined a significant main effect of session (F [2.3, 48.6] = 9.0; *p* < 0.001; η^2^ = 0.30; Huynh-Feldt corrected) and a significant main effect of flanker configuration (F [1, 21] = 6.1; *p* = 0.022; η^2^ = 0.22). The interaction between session and flanker configuration (F [2.4, 49.9] = 2.2; *p* = 0.11; η^2^ = 0.10; Huynh-Feldt corrected) was not significant. Further, the main effect of optotype was not significant (F [1, 21] = 0.008; *p* = 0.93; η^2^ < 0.001), as well as the interactions between session and optotype (F [2.3, 48.6] = 1.0; *p* = 0.38; η^2^ = 0.05; Huynh-Feldt corrected) and among session, flanker configuration, and optotype (F [2.4, 49.9] = 0.39; *p* = 0.71; η^2^ = 0.02; Huynh-Feldt corrected). Additionally, we probed the learning effect on the anisotropy further by calculating difference scores between radial and tangential target-to-flanker distances for each session and group, which are depicted in Figure 3C. We conducted a repeated measures ANOVA on the difference scores with the within-subjects factor session (session 1–4) and the between-subjects factor optotype (Landolt-C versus Tumbling-E). The ANOVA revealed no significant main effect of session (F [2.4, 49.9] = 2.2; *p* = 0.11; η^2^ = 0.10; Huynh-Feldt corrected), no significant main effect of optotype (F [1, 21] = 0.15; *p* = 0.70; η^2^ = 0.01) and no significant interaction between session and optotype (F [2.4, 49.9] = 0.39; *p* = 0.71; η^2^ = 0.02), but a post hoc test, that explored explicitly a trend over the four time points yielded a significant linear trend of session (F [1, 21] = 5.6; *p* = 0.027; η^2^ = 0.21).

To explore the training effects further, we also analyzed the development of the slope of the psychometric functions, which indicates the rate of change in performance as a function of target-flanker distance. The greater the slope, that is the steeper the psychometric functions, the more abruptly changes the performance rate of a subject from one target-flanker distance to the next larger one, thus giving a measure for the profile of the transition from crowding to no crowding. Supplementary Figure S2 shows the trends in the slope over the course of training. A repeated-measures ANOVA with the within-subjects factors session (sessions 1–4) and flanker configuration (radial versus tangential) and the between-subjects factor optotype (Landolt-C versus Tumbling-E) revealed a significant main effect of session (F [3, 63] = 36.8; *p* < 0.001; η^2^ = 0.64), a significant main effect of flanker configuration (F [1, 21] = 34.8; *p* < 0.001; η^2^ = 0.62) and a significant interaction between session and flanker configuration (F [3, 63] = 3.1; *p* = 0.031; η^2^ = 0.13). There was no significant main effect of optotype (F [1, 21] = 0.72; *p* = 0.405; η^2^ = 0.03), as well as no significant interactions session times optotype (F [3, 63] = 0.80; *p* = 0.499; η^2^ = 0.04), flanker configuration times optotype (F [1, 21] = 0.05; *p* = 0.832; η^2^ = 0.002) and session times flanker configuration times optotype (F [3, 63] = 0.28; *p* = 0.836; η^2^ = 0.01).

### Transfer of learning to the other optotype

To investigate transfer effects of learning from one optotype to the other we administered *t*-tests for the target-to-flanker distances (62.5% thresholds) between session 1 and session 5, as well as between session 4 and session 5, separately for each optotype and flanker configuration. The results are given in Table 2. They show that a complete transfer of learning is observed from training optotype Landolt-C to optotype Tumbling-E, with target-to-flanker distances in session 5 even significantly lower than for Landolt-C in session 4. On the other hand, no transfer of learning is observed from training optotype Tumbling-E to optotype Landolt-C. Target-to-flanker distances in session 5 for the Landolt-C are not significantly different from those obtained in session 1 for the Tumbling-E.

**Table 2.** Transfer effects, probed with *t*-tests on the 62.5% thresholds between day 1 and day 5, as well as between day 4 and day 5, separately for the two training groups. *P* values were Bonferroni-adjusted for multiple testing.

		Landolt-C			Tumbling-E		
	Training of	T	df	*p* value	T	df	*p* value
**Radial**	Day 1 vs. day 5	4.3	10	**0** **.002**	1.5	11	0.306
	Day 4 vs. day 5	4.2	10	**0** **.004**	−4.1	11	**0** **.004**
**Tangential**	Day 1 vs. day 5	4.2	10	**0** **.004**	−0.25	11	1.00
	Day 4 vs. day 5	3.4	10	**0** **.014**	−1.8	11	0.208

Supplementary Table S1 shows the transfer effects as evaluated by *t*-tests for the slopes of the psychometric functions. There was a complete transfer effect in regard to the slopes in the radial condition for the group who trained with the Landolt-C, and a partial transfer effect for the group who trained with the Tumbling-E. In the tangential condition, no significant transfer effects could be observed for both optotypes for the slopes.

## Discussion

In this experiment, we aimed to replicate the findings of Malania et al. (2020) with the same optotype (Landolt-C) as well as a different optotype (Tumbling-E) as target stimuli and explored the effect of training on the radial-tangential anisotropy in crowding. Additionally, we probed transfer effects of learning between the two optotypes. Here, we discuss the findings in light of the following aspects: the nature of the learning effect on crowding, the effect of learning on the radial-tangential anisotropy, and the transfer of learning to another optotype.

### Effect of learning on crowding

Several studies have shown that the strength and extent of crowding could be reduced by training (e.g. Chung, 2007; Hussain et al., 2012; Sun et al., 2010). In this, and similarly in our previous study (Malania et al., 2020), we aimed to measure the learning effect as a reduction of the crowding zone, here represented by the target-to-flanker distance that leads to 62.5% of correct responses (the 62.5% threshold). As such, the procedure is similar to the training phase of Hussain et al. (2012), who adjusted a spacing threshold of 79% correct between the target letter and the flanking letters by a staircase procedure and who could show that the spacing threshold decreased with training. Here, we could also show that the 62.5% threshold of the target-to-flanker distance significantly decreased with training (see Figure 3), an effect that was observed for both optotypes and was more pronounced in the radial flanker condition. As such, we would conclude that the crowding zone as the target-to-flanker distance, where the target letter could be recognized, can be reduced by training. Additionally, we analyzed the trends in the slopes of the psychometric functions with training. Figure S2 shows the tendency of the slope to increase over time. This gives us a measure for the transition from crowding to no crowding. After training, the slope of this transition became steeper. That means that with training less target-to-flanker spacing was necessary to achieve the same performance gain as before training. This effect was also observed for both optotypes and was more pronounced in the radial condition. As Supplementary Figure S1 shows, most learning occurred for flanker spacings between 1.5 degrees and 3 degrees visual angle. There is almost no performance gain for flanker spacings smaller than that, although this is also dependent on the optotype (see below). Critically, it has to be noted that other factors could also play a role in performance improvements in this task that could not be addressed properly by our paradigm and analysis (e.g. a change in response criteria). Particularly, our 4AFC-task on the orientation of the target stimulus, which was different from the flanker stimuli (closed rings and squares), did not allow participants to report the flanker instead of the target. At the same time, target and flanker could not be mixed up with each other. It has been shown in the literature that the strength of crowding can be reduced, if the naming of the flankers is not a response option in the task (e.g. Reuther & Chakravarthi, 2020; Rosenholtz, Yu, & Keshvari, 2019). As such, our paradigm cannot clearly distinguish between these two possible effects of training: a reduction of the crowding zone or an improvement in the ability of the subjects to exclude the flanker stimuli from their decision making. Another aspect that should be considered is the role of feedback in this perceptual learning task. Former studies on perceptual learning of a crowding task often used trial-by-trial feedback (e.g. Chung, 2007; Hussain et al., 2012; Sun et al., 2010; Xiong et al., 2015; Yashar et al., 2015). Herzog and Fahle (1997) had systematically explored the effects of different kinds of feedback (trial-by-trial feedback, block-wise feedback, and manipulated feedback) in a perceptual learning task and showed that trial-by-trial feedback led to a larger improvement in performance than the no feedback condition. In a former study on perceptual learning of a coherent motion task, we found that trial-by-trial feedback promoted learning especially at medium-to-low levels of difficulty, whereas it appeared to have rather aversive effects on learning at the highest levels of difficulty (Goldhacker, Rosengarth, Plank, & Greenlee, 2014). Other studies, including those on task irrelevant learning, also showed that learning could occur without explicit external reinforcements (external feedback; e.g. Ball & Sekuler, 1982; Karni & Sagi, 1991; Watanabe, Náñez, & Sasaki, 2001). Here, we used only block feedback and no explicit feedback after each trial, and, interestingly, improvements were also more pronounced in the medium-to-low levels of difficulty. But because we did not explore the effects of trial-by-trial feedback in this task, we do not know how this might have changed the learning curves of the subjects. Further research would be needed to determine the exact role of feedback in perceptual learning of crowding.

### Effect of learning on the radial-tangential anisotropy

In our previous study (Malania et al., 2020), where participants trained on a crowding task on Landolt-C targets with radially and tangentially arranged flankers, we found a significant reduction in the radial-tangential anisotropy in the course of training. Here, we aimed to replicate this finding in another subject group and additionally for training on another optotype (Tumbling-E). Similarly to Malania et al. (2020), we adjusted cumulative normal distributions to the data and calculated the target-to-flanker distance thresholds (as measures of the crowding zone), for which we predicted that they should decrease with learning. As Figure 3 shows, we found a decrease of the crowding zone over time that was more pronounced in the condition with radially arranged flankers, leading to a decrease of the difference scores between crowding zones in the radial and tangential condition (see Figure 3C). Although a significant linear trend could be observed for the difference scores, the interaction effect between session and flanker configuration failed to reach significance in the conducted repeated-measures ANOVA. On the other hand, the repeated-measures ANOVA conducted directly on the hit rates (including the flanker spacings as a factor; see also Supplementary Figure S1) revealed a significant interaction between session and flanker configuration. As such, the results show a trend toward reduction of the radial-tangential anisotropy with training, without clear evidence for it to disappear completely – at least not during the four training sessions applied here. Although the radial-tangential anisotropy can be considered a rather stable and consistent feature of crowding (e.g. Greenwood, Szinte, Sayim, & Cavanagh, 2017), Chung (2013) found a reduction of the anisotropy in patients with central vision loss at those patients’ PRL in the peripheral visual field that functions as a kind of a pseudo-fovea. This effect was ascribed to the extensive usage of that area in those patients’ daily vision.

### Transfer of learning to another optotype

Another research question of this study was, if learning of a crowding task with a certain optotype could transfer to a different optotype. Here, we chose the two optotypes Landolt-C and Tumbling-E that are well established optotypes used in visual acuity tests (e.g. Bach, 1996). Participants trained the crowding task either on the Landolt-C or the Tumbling-E over four sessions and switched to the other optotype in session 5. Interestingly, we found a complete transfer of training from the Landolt-C optotype to the Tumbling-E optotype, but not vice versa (see Figure 3, Table 2, as well as Supplementary Figure S1). Moreover, participants who trained the Landolt-C task showed improved performance when switching to Tumbling-E, whereas participants who trained with Tumbling-E achieved worse results after switching to Landolt-C. This result suggests that the Tumbling-E condition was overall easier to perform than the Landolt-C condition. Because we knew from our earlier study on perceptual learning of crowding with a Landolt-C target stimulus (Malania et al., 2020) that a substantial amount of learning usually occurs already in the first session of performing the crowding task, it was not possible to test each group in the other optotype pretraining without risking to lose the participants’ “pretraining performance status” in that other optotype that we aimed to test in session 5. For that reason, we decided against a pretest in the other optotype, but analyzed differences in performance on each optotype during the learning phase of each group. As Table 1 and Supplementary Figure S1 show for the no-flanker control condition, there was no significant difference between the two optotypes and they were comparably easy to recognize, when they were presented isolated at the chosen eccentricity of 6.5 degrees visual angle in the upper right quadrant. In addition, learning effects, as measured by the reduction of target-to-flanker distances (62.5% thresholds; see Figure 3) and as development of percent-correct values on each flanker spacing (see Figure S1), over 4 days of training did not differ significantly between the two optotypes. Neither the repeated-measures ANOVA on the 62.5% thresholds nor the repeated-measures ANOVA on the percent-correct values revealed any significant main effect of optotype or significant interaction effects with the factor optotype. As such, we did not gain explicit evidence for one optotype to be distinctly easier to be recognized than the other, neither in isolation nor in the crowded condition. Descriptively, it can be seen from Figure S1 that the group who trained with the Tumbling-E on average exhibited performance gains in the radial condition on medium-level flanker spacings (1.5 degrees to 2.0 degrees) earlier (session 2) than the group who trained with the Landolt-C, whereas both groups showed comparable learning progress at greater flanker spacings. Further, the Tumbling-E group could raise their performance levels at the smallest flanker spacing 0.75 degrees in sessions 3 and 4, which was not the case for the Landolt-C group. What could be reasons for the slight advantage in recognizing Tumbling-Es over Landolt-Cs of the same size? Already Kaufmann and Decker (1986) asserted that, to achieve the same performance, the size of the Tumbling-E should be only 87% of that of the Landolt-C. On the other hand, we did not find clear evidence for performance differences on the two optotypes when presented in isolation, where we observed ceiling effects (see Table 1), which makes it more probable that the target-flanker-constellations offer possible explanations for differences in learning progress. According to Huckauf and Nazir (2007) and Yeotikar et al. (2013), learning effects in crowding tasks are often specific for the target-flanker-configuration trained. In this experiment, we used different flankers for Landolt-C (closed rings) and Tumbling-E (closed squares with a crossbar), which were chosen to appear comparably similar to their respective targets. Possibly, the target-flanker-configuration Landolt-C plus closed rings led to specific adaptation effects in the sensitive neurons that could help subsequently to differentiate between Tumbling-E and squared flankers better than vice versa. The Landolt-C task might challenge the visual system more to fine-tune gap detection, which as a consequence could result in better transfer to other stimuli. Flanker complexity and target-flanker similarity could also play a role. As Bernard and Chung (2011) have shown, flanker complexity can increase the strength of crowding. According to the perimetric complexity introduced by Pelli, Burns, Farell, and Moore-Page (2006), for the flankers in our study, complexity should be higher for the closed squares with a crossbar chosen for the Tumbling-E targets than the closed ring stimuli chosen as flankers for the Landolt-C targets. As such, the crowding effect in the Tumbling-E condition should actually have been greater, which would not be in line with our results. Several studies have also shown that target-flanker-similarity increases the strength of crowding (e.g. Bernard & Chung, 2011; Cheung & Cheung, 2017; Freeman, Chakravarthi, & Pelli, 2012; Kooi, Toet, Tripathy, & Levi, 1994). In our study, we aimed to keep target-flanker similarity equal for both conditions, but it might be that the ring flankers were more similar to the target Landolt-C than the squares with crossbar to the target Tumbling-E. As such, it would have been easier to distinguish the target Tumbling-E from the flankers than the Landolt-C. Less crowding in the Tumbling-E condition induced by less target-flanker similarity could possibly explain the slightly better learning progress in that condition. Despite these differences in overall difficulty between the two optotypes their learning curves largely overlap, which makes it unlikely that the observed transfer effects from Landolt-C to Tumbling-E only occurred due to different overall difficulty. On the other hand, the lack of a learning transfer from Tumbling-E to Landolt-C may be caused by the Landolt-C target-flanker-constellation exhibiting more crowding. In any case, these results show that transfer effects in learning of crowding are largely dependent on the exact configuration of the training and test stimuli, which will have implications for any practical use of such training procedures (e.g. with the aim to improve reading abilities in patients with central vision loss). Further research that tests training and transfer effects not only on optotypes but also on other letters and normal text will be necessary to gain further insight into that issue.

## Conclusions

The aim of this study was to contribute to the knowledge about the capacity of peripheral vision to improve by training. This is particularly relevant for patients with central vision loss, as they are reliant on their peripheral visual fields for daily tasks like reading. Transfer of learning is an important issue in this context, because patients would benefit most by perceptual training in their daily lives, if improvements are not specific to the trained stimulus configurations, but rather generalize to other objects and tasks as well. Here, we could show that the transfer effects of training on a crowding task were largely reliant on the specific target-flanker constellation used. Further research is therefore needed to identify those target-flanker constellations with the most promising transfer effects to other stimuli.

## Supplementary Material

Supplement 1

Supplement 2
