# Supplementary material for: Perceptual learning of a crowding task: Effects of anisotropy and optotype
Source: J Vis. 2021 Oct 21;21(11):13. doi: 10.1167/jov.21.11.13 (PMC8543403; doi:10.1167/jov.21.11.13)
Supplement: Supplement 2 [file jovi-21-11-13_s002.pdf]

Supplementary Material

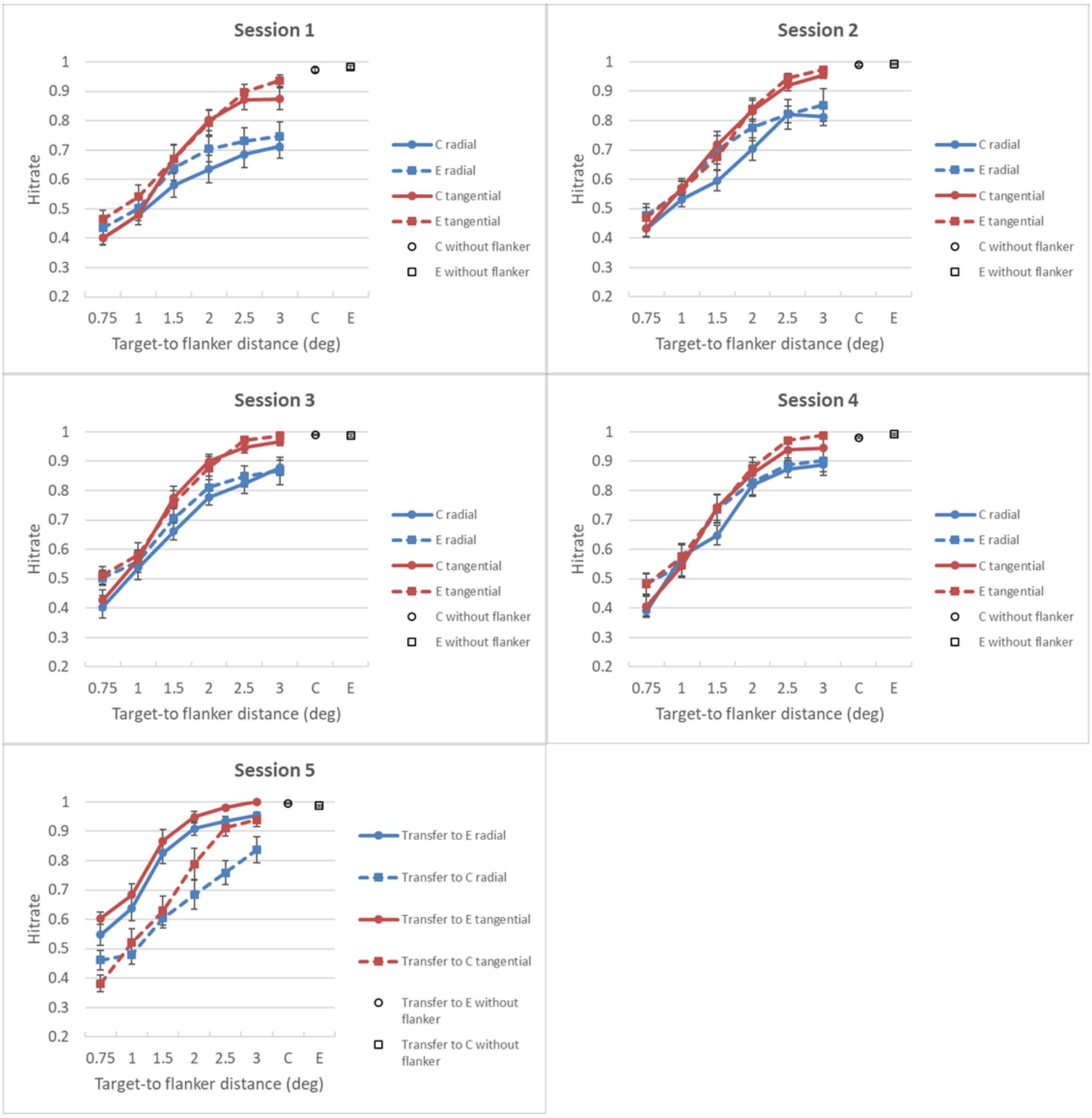

Figure S1: Mean percent correct values (+/- 1 SE) over the training period (sessions 1-4), as well as for the transfer to the other optotype in session 5, as a function of the presented target-to-flanker distances. Results for the radial flanker configuration are shown in blue, results for the tangential flanker configuration are shown in red. Solid lines and circles mark the results for the group who trained with Landolt-Cs and switched to Tumbling-Es in the fifth session, dashed lines and squares mark the results for the group who trained with Tumbling-Es and switched to Landolt-Cs in the fifth session. Additionally, the percent correct values (+/- 1 SE) for the optotypes Landolt-C and Tumbling-E presented in isolation are shown for each session and group.

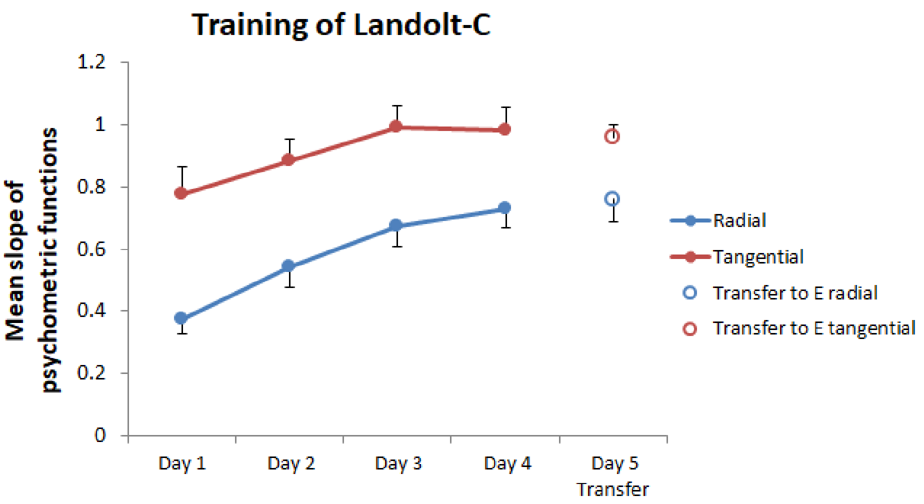

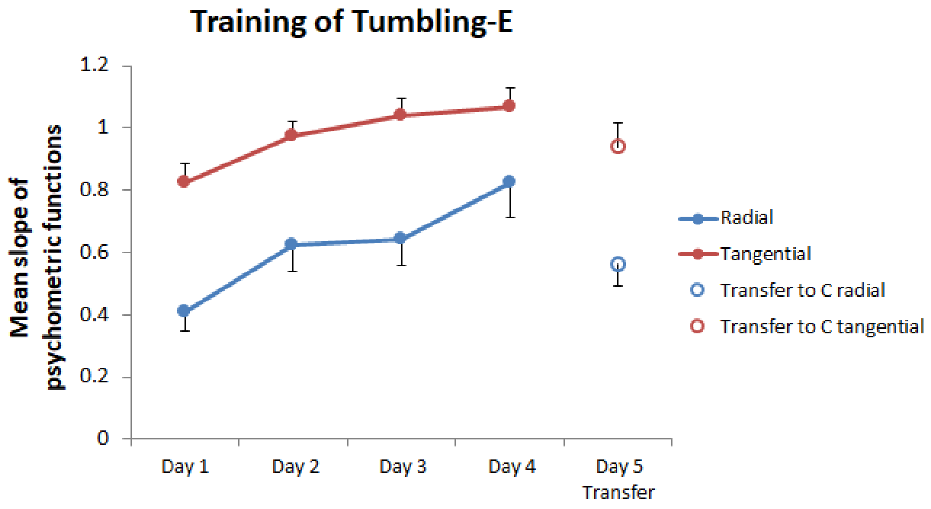

Figure S2: Mean slopes (+/- 1 SE) of the psychometric functions of each participant over the training period of four days, as well as for the transfer to the other optotype on day 5. Upper panel: Group who trained with Landolt-C and transferred to Tumbling-E on day 5. Lower Panel: Group who trained with Tumbling-E and transferred to Landolt-C on day 5. The data for the radial flanker configurations are shown in blue, the data for the tangential flanker configurations are shown in red.

Table S1: Transfer effects, probed with t-Tests on the slope of the psychometric functions between Day 1 and Day 5, as well as between Day 4 and Day 5, separately for the two training groups. P-values were Bonferroni-adjusted for multiple testing.

	**Training of**	**Landolt-C**			**Tumbling-E**		
		**T**	**df**	**p**	**T**	**df**	**p**
**Radial**	Day 1 vs. Day 5	-4.5	10	**.002**	-3.3	11	**.014**
	Day 4 vs. Day 5	-.33	10	1.00	3.6	11	**.008**
**Tangential**	Day 1 vs. Day 5	-2.1	10	.132	-1.5	11	.344
	Day 4 vs. Day 5	.27	10	1.00	1.5	11	.344
